# Impact of the metal core on the electrochemiluminescence of a pair of atomically precise Au_20_ nanocluster isomers

**DOI:** 10.1038/s42004-023-00907-4

**Published:** 2023-05-31

**Authors:** Shuang Chen, Ying Liu, Kaiyang Kuang, Bing Yin, Xiaojian Wang, Lirong Jiang, Pu Wang, Yong Pei, Manzhou Zhu

**Affiliations:** 1 Institutes of Physical Science and Information Technology, Anhui University, Hefei, Anhui 230601 PR China; 2 Centre for Atomic Engineering of Advanced Materials, Anhui University, Hefei, Anhui 230601 PR China; 3 Key Laboratory of Structure and Functional Regulation of Hybrid Materials of Ministry of Education, Anhui University, Hefei, Anhui 230601 PR China; 4 Department of Chemistry and Anhui Province Key Laboratory of Chemistry for Inorganic/Organic Hybrid Functionalized Materials, Anhui University, Hefei, Anhui 230601 PR China; 5 Department of Chemistry, Xiangtan University, Xiangtan, Hunan 411105 PR China; 6 Key Laboratory of Environmentally Friendly Chemistry and Applications of Ministry of Education, Xiangtan University, Xiangtan, Hunan 411105 PR China

**Keywords:** Structural properties, Synthesis and processing, Nanoparticles, Optical materials

## Abstract

Although the electrochemiluminescence (ECL) of metal nanoclusters has been reported, revealing the correlation between structure and ECL at an atomic level is highly challenging. Here, we reported the impact of the metal core of Au_20_(SAdm)_12_(CHT)_4_ (Au_20_-AC for short; SAdm = 1-adamantanethiolate; CHT= cyclohexanethiol) and its isomer Au_20_(TBBT)_16_ (TBBT = 4-tert-butylthiophenol) on their solution-state and solid-state electrochemiluminescence. In self-annihilation ECL experiments, Au_20_-AC showed a strong cathodic ECL but a weak anodic ECL, while the ECL signal of Au_20_(TBBT)_16_ was weak and barely detectable. Density functional theory (DFT) calculations showed that the Au_7_ kernel of [Au_20_-AC]^-^ is metastable, weakening its anodic ECL. Au_20_-AC in solution-state displayed an intense co-reactant ECL in the near-infrared region, which is 7 times higher than that of standard Ru(bpy)_3_^2+^. The strongest solid-state ECL emissions of Au_20_-AC and Au_20_(TBBT)_16_ were at 860 and 770 nm, respectively — 15 nm red-shifted for Au_20_-AC and 20 nm blue-shifted for Au_20_(TBBT)_16_, compared to their corresponding solid-state photoluminescence (PL) emissions. This work shows that ECL is significantly affected by the subtle differences of the metal core, and offers a potential basis for sensing and immunoassay platforms based on atomically precise emissive metal nanoclusters.

## Abstract (web summary)

Metal nanoclusters have shown great promise as electrochemiluminescence (ECL) luminophores, but understanding the relationship between ECL and atomic structure is highly challenging. Here, the ECL of nanocluster isomers Au_20_(SAdm)_12_(CHT)_4_ and Au_20_(TBBT)_16_ is studied, giving insight into structure–property relationships.

## Introduction

Electrogenerated chemiluminescence entails the generation of electronically excited species at electrode surfaces which emit light upon their relaxation to a lower-level state. ECL is a highly sensitive and selective analytical technique, with a low detection limit in absence of background light^1–9^. Metal nanoclusters have been demonstrated to be excellent ECL luminophores due to their high intensity and efficiency^10–18^. Determined structure and fascinating properties of metal nanoclusters provide important insights for structure-property correlations and important guidance for designing functional nanomaterials^19–22^. Although progress of ECL of metal nanocluster has been made, the influence factors of ECL of metal nanoclusters have been unrevealed. Structural isomers of metal nanocluster are ideal models for understanding the effect of different structural moieties on ECL properties^23–26^. Thus far, only once case of correlation between structure and ECL property of metal nanocluster has been reported^27^, yet whether the different influencing factors affect the behavior of ECLs in solution is still unknown. Recently, solid-state ECL (SSECL) has been developed after the aggregation-induced ECL (AIECL) proposed^28–33^. Aggregation-induced emission (AIE) materials provide the possibility for AIECL. In metal nanocluster, Xie et al. revealed gold nanoclusters with long Au-SR motifs could generate strong emissions due to the aggregation of motifs on the metal cores^32^, which is desirable for the ECL of metal nanocluster in solid state.

Herein, we report the preparation and structure of Au_20_(SAdm)_12_(CHT)_4_ (abbrev. Au_20_-AC below, SAdm = 1-adamantanethiol, CHT = cyclohexanethiol), whose structure is similar to that of Au_20_-Iso1, as predicted by Pei and co-workers^34^; Au_20_-AC contain one Au_7_ core, two Au_2_(SR)_3_, one Au_3_(SR)_4_ and one long Au_6_(SR)_6_ motif. We explored self-annihilation and coreactant ECLs of Au_20_-AC and its isomer Au_20_(TBBT)_16_ (TBBT = 4-tert-butylthiophenol, reported by Jin et al.^35^) in solution and solid states. Electrochemistry, ECL, PL and density functional theory (DFT) simulation have been employed to establish a correlation between structure and ECL performance in different states. It is observed that Au_20_-AC displayed an intense cathodic ECL and a weak anodic ECL, while Au_20_(TBBT)_16_ showed weak cathodic and anodic ECLs in self-annihilation pathway. DFT calculations indicate that the anion radical is destabilized due to metal core distortion for Au_20_-AC, leading to weak anodic ECL. ECL spectrum of Au_20_-AC in solution state is centered at 830 nm in the presence of TPrA. The oxidative reduction SSECL of both Au_20_ clusters in phosphate buffer solution (PBS, pH = 7.5) was observed in the near-infrared region. Overall, our correlation of the structure of these Au metal nanocluster isomers with their ECL performance constitutes a possible approach towards the design of intense ECL emitters and the development of associated detection platforms^6,36–38^.

## Results and discussion

### Structure and characterization of Au_20_-AC

Au_20_-AC was formed by ligand etching Au_18_(CHT)_14_ nanoclusters with HSAdm^39^ and purified by TLC. As shown in Supplementary Fig. 1, three bands corresponding to Au_20_-AC, Au_16_ and Au_21_ nanoclusters from top to bottom, were observed. The UV-vis absorption spectra of these three nanoclusters are shown in Supplementary Fig. 2. Rhombic crystals of Au_20_-AC could be obtained within 2~3 days using a mixture of methanol and CH_2_Cl_2_.

**Fig. 1:**
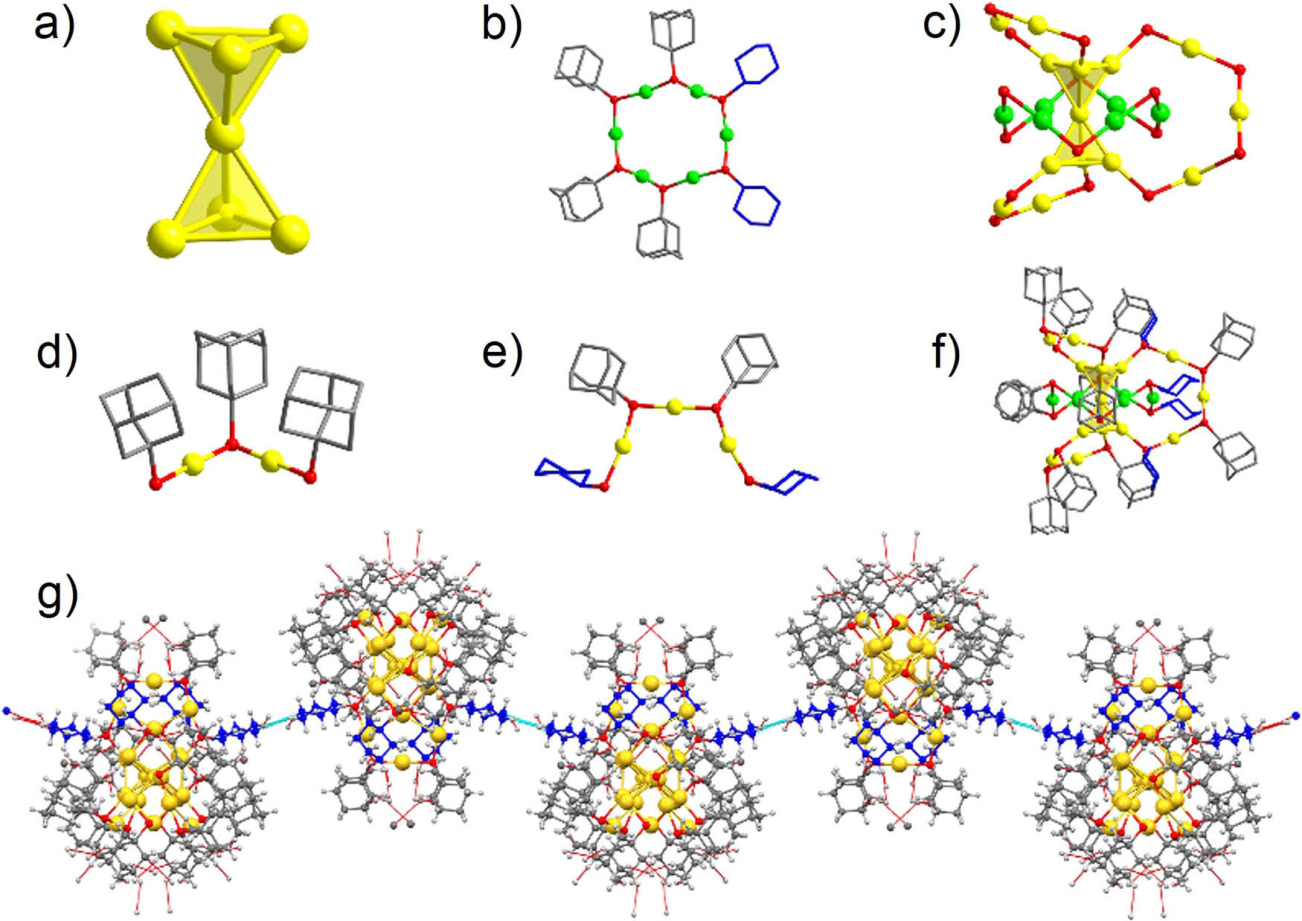
Total structure of Au_20_-AC. **a** The di-tetrahedral Au_7_ kernel. **b** The circular Au_6_(SAdm)_4_(CHT)_2_ motif. **c** The structure of kernel and motifs in Au_20_(SAdm)_12_(CHT)_4_. **d** The dimeric Au_2_(SAdm)_3_ motif. **e** The trimeric Au_3_(SAdm)_2_(CHT)_2_ motif. **f** The total structure of Au_20_(SAdm)_12_(CHT)_4_, all H atoms are omitted for clarity. **g** The arrangement of cyclohexane rings in a head-to-head pattern in neighboring CHT of Au_20_-AC. (Color labels: green, yellow = Au; red = S; grey, blue = C; white = H).

The structure of Au_20_-AC was determined by single-crystal X-ray diffraction (Supplementary Data 1), which revealed that its crystals adopt the monoclinic space group C2/c. Full details are presented in Fig. 1 and Supplementary Table 1. Structurally, Au_20_-AC is almost identical to the predicted Au_20_-Iso1, and contains a Au_7_ kernel comprised of two fused tetrahedra (Fig. 1a) arranged linearly through a common Au atom, different to the twisted tetrahedra found in Au_20_(TBBT)_16_. The average Au−Au bond length in the Au_7_ kernel of Au_20_-AC (2.730 Å) is slightly (0.40%) longer than that in Au_20_(TBBT)_16_ (2.719 Å). Au_2_(SR)_3_, Au_3_(SR)_4_ and circle-like Au_6_(SR)_6_ motifs were observed to cap the core tetrahedra (Fig. 1d, e, b). Interestingly, the mixed thiol ligands HSAdm and CHT comprise the Au_3_(SR)_4_ and Au_6_(SR)_6_ motifs, forming Au_3_(SAdm)_2_(CHT)_2_ and Au_6_(SAdm)_4_(CHT)_2_ motifs. The energy of Au_20_-Iso1 was predicted to be comparable to or even lower than that of Au_20_(TBBT)_16_^34^. The use of mixed ligands instead of the more usual single thiol ligand might facilitate the experimental preparation of Au_20_-Iso1. Regarding the Au_20_-AC superstructure, the cyclohexane units present in the Au_6_(SAdm)_4_(CHT)_2_ motifs adopt a chair configuration and are arranged in a head-to-head pattern due to the non-covalent interactions of C…H and H…H (Fig. 1g), contributing to the high stability of Au_20_-AC in the aggregated state.

The composition of Au_20_-AC was further confirmed by electrospray ionization mass spectrometry, X-ray photoelectron spectroscopy and thermogravimetric analysis. Cesium acetate (CsOAc) was added, to form adducts. As shown in Supplementary Fig. 3, the electrospray ionization mass spectra depicts a series of peaks, each separated by 52 Da and corresponding to a series of different Au_20_-AC moieties. [Au_20_(C_10_H_15_S)_16-n_(C_6_H_11_S)_n_ + Cs]^+^ (*n* = 4~9) containing HSAdm and CHT ligands in different combinations, reflecting their dynamic equilibrium in the solution state. However, the crystal state Au_20_-AC was determined by single crystal diffraction to contain 12 HSAdm and 4 CHT ligands. In mass spectrometry, the isotopic peaks of [Au_20_(SAdm)_12_(CHT)_4_ + Cs]^+^ are consistent with the simulated spectra (Supplementary Fig. 3). The composition of Au_20_-AC was also confirmed by X-ray photoelectron spectrum (Supplementary Fig. 4) and thermogravimetric analysis (Supplementary Fig. 5).

### Photoluminescence and voltammetry analysis of Au_20_ isomers

**Fig. 2:**
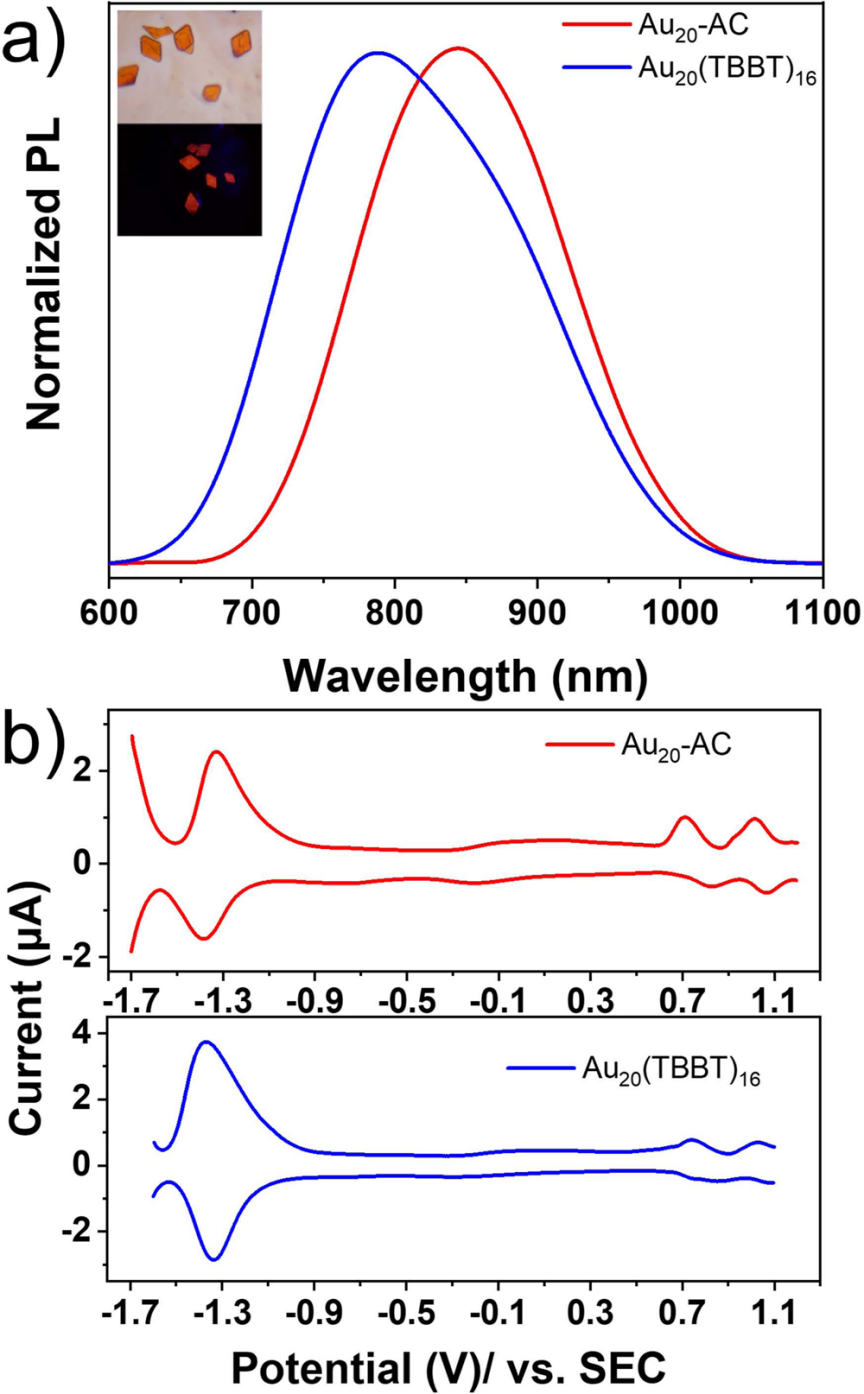
Normalized PL of solid Au_20_-AC and Au_20_(TBBT)_16_ and square wave voltammetry (SWV) of Au_20_-AC and Au_20_(TBBT)_16_. **a** Normalized PL of solid Au_20_-AC and Au_20_(TBBT)_16_. Inset: The crystal of Au_20_-AC under visible light (top) and ultraviolet light (bottom), respectively. **b** SWV of 0.015 mM Au_20_-AC and Au_20_(TBBT)_16_ in 1:1 TOL: ACN with 0.1 M TBAP. Pt disk was used as working electrode, Pt foil and SCE were used as counter and reference electrode, respectively.

Au_20_-AC and Au_20_(TBBT)_16_ in solid state were intensely photoluminescent. Figure 2a shows the PL spectra of solid Au_20_-AC (red line) and Au_20_(TBBT)_16_ (blue line); the peaks are at 845 and 790 nm, respectively. A solution of Au_20_-AC was non-fluorescent at the same excitation wavelength, reflecting its strong AIE behaviour (Supplementary Fig. 6). In the solution state, van der Waals forces drive the rotation of molecules until their surface motifs establish stable interactions with neighbouring nanoclusters; the free rotation of the ligands and any dissociation/association of the thiol ligands results in the loss of non-radiative energy^40^. Accordingly, Au_20_-AC was barely luminescent in solution. However, the ligands of aggregated and crystallized nanoclusters are fixed. This restriction of intramolecular rotation (RIR) and suppression of any disaggregation and association processes inhibits the non-radiative decay of excited states, resulting in intense AIE in the solid state. The ligands of Au_20_-AC are non-aromatic and thus unable to π–π stack, but their long-range interaction (between unit cells) may still affect their PL^40^. Aggregated Au_20_-AC showed a longer PL lifetime (2.19 μs) than aggregated Au_20_(TBBT)_16_ (624 ns) (Supplementary Figs. 7 and 8).

Cyclic voltammetry (CV) studies of Au_20_-AC and Au_20_(TBBT)_16_ were carried out by scanning between −1.7 and 1.2 V vs. SCE at a scan rate of 0.1 V s^−1^ (Supplementary Figs. 9 and S10). The square wave voltammetry (SWV) of Au_20_-AC and Au_20_(TBBT)_16_ was tested in a 1:1 solution of toluene (TOL)/acetonitrile (ACN) with 0.1 M tetrabutylammonium perchlorate (TBAP) as the electrolyte. As shown in Fig. 2b, two quasi-reversible oxidation waves at E_Ox_ = 0.71 V and 1.01 V were observed (Fig. 2b, red line in top panel). These correspond to the Au_20_-AC being oxidized consecutively to [Au_20_-AC]^1+^ and then [Au_20_-AC]^2+^. Au_20_-AC shows one multi-electron reduction wave at E_Re_ = −1.19 V, which indicates an irreversible reduction process in Au_20_-AC. Two oxidation peaks of Au_20_(TBBT)_16_ at 0.74 V and 1.02 V and a multi-electron reduction peak at −1.13 V were observed (Fig. 2b, blue line in down panel). All these redox processes of Au_20_(TBBT)_16_ are quasi-reversible. The potential differences between the first reduction and oxidation peak of Au_20_-AC and Au_20_(TBBT)_16_ were 2.10 and 2.07 V. The surface ligands and motifs of Au_20_-AC lowered its reduction and oxidation potentials compared to those of Au_20_(TBBT)_16_. The propensity of these nanoclusters to gain and lose electrons is related to their HOMO–LUMO gap and ligand-metal and metal-metal electronic coupling/interaction properties^41^.

To rationalize the electrochemical properties of the gold clusters, DFT/PBE calculations were performed—the HOMO energies of Au_20_-AC and Au_20_(TBBT)_16_ were calculated to be −4.23 eV and −4.41 eV, respectively (Supplementary Fig. 11). This is consistent with the lower oxidation potential of Au_20_-AC compared to Au_20_(TBBT)_16_ (0.71 V vs 0.74 V); since the oxidation process involves removing an electron from the HOMO energy level, the Au_20_-AC cluster with the higher HOMO energy level will lose an electron more easily compared to Au_20_(TBBT)_16_, whose lower HOMO energy level (−4.41 eV) is consistent with its higher relative oxidation potential (0.74 V). On the contrary, the LUMO energy level will acquire an electron during reduction, thus the Au_20_-AC cluster with a higher LUMO energy level (−2.38 eV) will have more difficulty in obtaining an electron, resulting in a relatively low reduction potential (−1.19 V). The Au_20_(TBBT)_16_ cluster with a lower LUMO energy level (−2.74 eV) will have a relatively high reduction potential (−1.13 V). The HOMO-LUMO gaps of the Au_20_-AC and Au_20_(TBBT)_16_ clusters are 1.85 eV and 1.67 eV, respectively. The DFT calculations are in good agreement with the experiments.

### ECL of Au_20_ isomers in solution state and DFT theoretical analysis

**Fig. 3:**
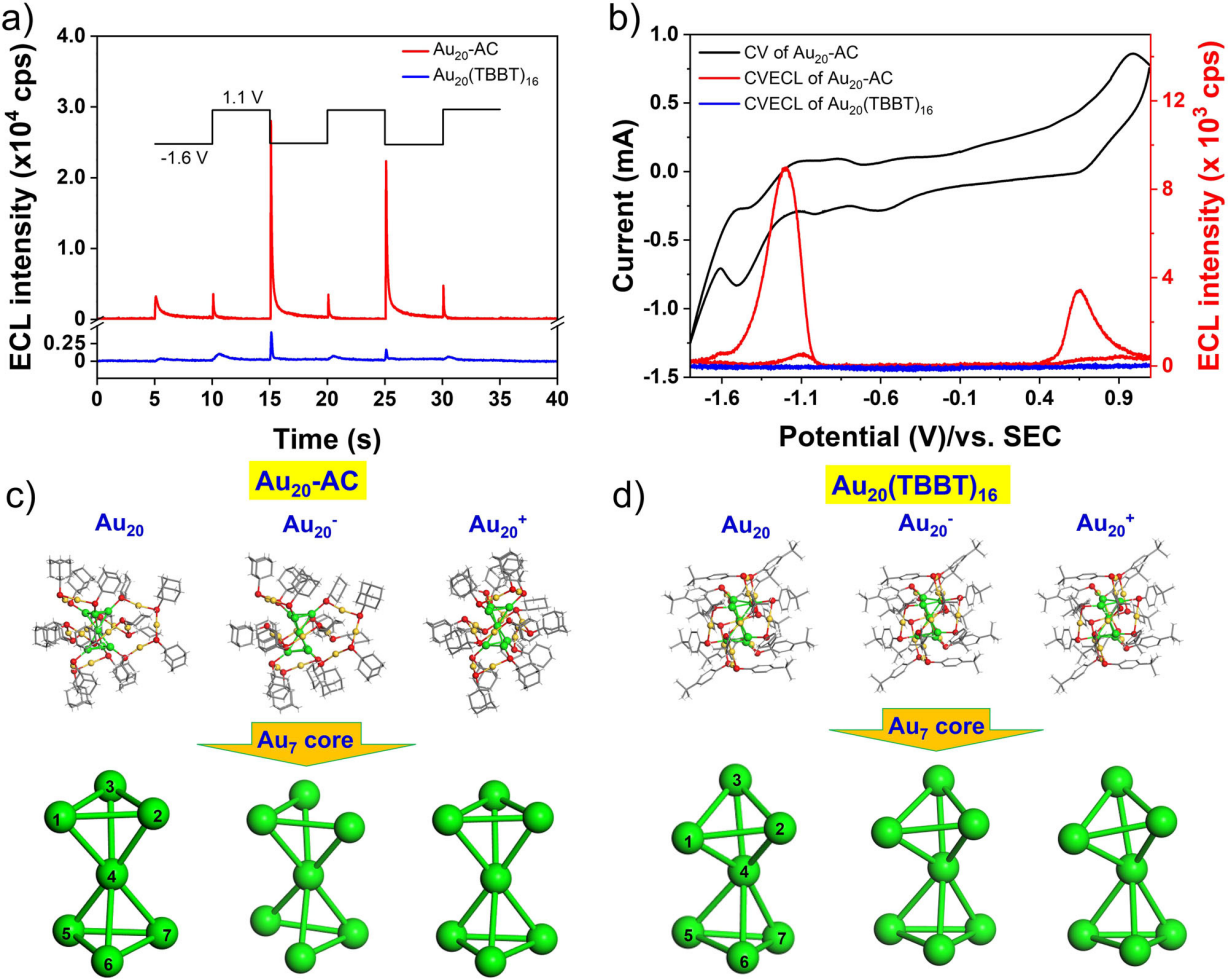
ECL of Au_20_-AC and Au_20_(TBBT)_16_ under potential step and scanning conditions and calculated stability of different valence states of Au_20_-AC and Au_20_(TBBT)_16_ radical. **a** Potential step self-annihilation ECL of Au_20_-AC and Au_20_(TBBT)_16_. The concentration of nanocluster is 0.015 mM in Tol/ACN (1:1) with 0.1 M TBAP electrolyte. The potential was set between −1.6 and 1.1 V and stepped cyclically, each potential was held for 5 s, and no potential was applied in the first and final 5 s. Pt mesh was used as working electrode, Pt foil and SCE were used as counter and reference electrode, respectively. **b** Self-annihilation ECL-voltage curves from −1.6 V to 1.1 V of Au_20_-AC and Au_20_(TBBT)_16_ in solution state. Initial scan to negative potential direction. The 3^rd^ cycle data was shown. **c**, **d** Theoretical calculation of structures of different valence states of Au_20_-AC and Au_20_(TBBT)_16_ radical. The corresponding bond length is shown in Supplementary Table 2.

The ECLs of both nanoclusters in solution state was studied in TOL/ACN (1:1) with Pt mesh and applied potential of between −1.6 V and 1.1 V. Figure 3a shows the step ECLs of both Au_20_ nanoclusters with negative and positive potentials alternately applied for three cycles. Overall, the ECL of Au_20_-AC is stronger than that of Au_20_(TBBT)_16_. Au_20_-AC shows intense ECL at −1.6 V (onset of 15 and 25 s) but weak ECL at 1.2 V (onset of 10, 20 and 30 s). In addition, the self-annihilation ECL of Au_20_-AC and Au_20_(TBBT)_16_ were explored in potential scanning experiment. As shown in Fig. 3b, both cathodic and anodic ECL were observed in the 3^rd^ cycle scanning potential for Au_20_-AC and the onset potential of reductive ECL of Au_20_-AC is at −1.0 V that is consistent with the onset potential of reduction peak in CV curve. No cathodic ECL signal was observed in the potential sweep of the first cycle because no cationic radicals were generated at this time (Supplementary Fig. 12). The ECL intensity of Au_20_-AC under consecutive potential scans decreased slightly with relative standard deviation (RSD) of 7.52% (Supplementary Fig. 13). While Au_20_(TBBT)_16_ display quite weak self-annihilation ECL at all potentials (Fig. 3b, blue curve).

To explore the influence factors of ECL performance of such two Au_20_ nanoclusters, we performed DFT calculations and studied the metal core and motif effects on their ECLs. As shown in Fig. 3c, d and Supplementary Table 2, both the Au2-Au3 and Au5-Au6 bond lengths in the Au_7_ kernel of the [Au_20_-AC]^-^ anion were longer than those in the neutral Au_20_-AC cluster (3.281 Å vs 2.810 Å and 3.050 Å vs 2.819 Å, respectively), reflecting their lower bond energies. There was no significant difference in the averaged Au-S distances in the core-shell and motif, indicating bare influence of different thiols and motifs on ECL performance (Supplementary Fig. 14 and Table 3). These bond length analyses and structural representations (Fig. 3c, d) indicate that the metal core of [Au_20_-AC]^-^ is distorted, reducing the overall stability of the [Au_20_-AC]^-^ and precluding its ability to react with cluster cations to form a strong anodic ECL signal (Fig. 3a, onset of 10, 20 and 30 s). The low stability of [Au_20_-AC]^-^ from theoretical simulations is consistent with the result obtained from voltammetric curves that [Au_20_-AC]^-^ is irreversible during the reduction process (Fig. 2b, top panel). However, more stable cluster radical cations can react with newly formed radical anions to form stronger cathodic ECL signals during the application of negative potential (Fig. 3a, onset of 15 and 25 s).

A possible self-annihilation ECL mechanism of the Au_20_ clusters is proposed in Supplementary Equation (1-4) (Supplementary Information Section 3); Au_20_ and Au_20_* denote the ground- and excited-states of the Au_20_ (including Au_20_-AC and Au_20_(TBBT)_16_) clusters, respectively. In the self-annihilation ECL process, negatively charged Au_20_^−∙^ and positively charged Au_20_^+∙^ cluster radicals are generated by electrode electron transfer reaction (Supplementary Equation (1) and (2)). Electrons or holes are transferred between cluster and the electrode surface. The generated radical cation and anion react and produce excited Au_20_* (Supplementary Equation (3)), which relax to the ground state and release the energy via photon emission (Supplementary Equation (4)). The self-annihilation ECL intensity is dependent on the stabilities of the Au_20_^−∙^ and Au_20_^+∙^ intermediates that are generated on the electrode upon application of a potential. Simultaneously, the transfer rate of the electrons and holes, the reaction capability of radical anions and cations, and the radiative efficiency of the excited state will affect the ECL intensity. Although the metastability of anodic and cationic radicals of Au_20_(TBBT)_16_ cluster were not observed in the DFT theoretical simulation, its ECL signal was barely observed in potential and scanning experiments. Thus, we studied the emission capability of the excited states of Au_20_-AC and Au_20_(TBBT)_16_. As shown in Supplementary Fig. 15 and Table 4, both excited nanoclusters displayed comparable emission capability. We speculate that the limited transfer rate of the electrons and holes and weak reaction capability between radical anions and cations may be responsible for the weak self-annihilation of Au_20_(TBBT)_16_.

### ECL efficiency of Au_20_-AC and Au_20_(TBBT)_16_ in solution state

**Fig. 4:**
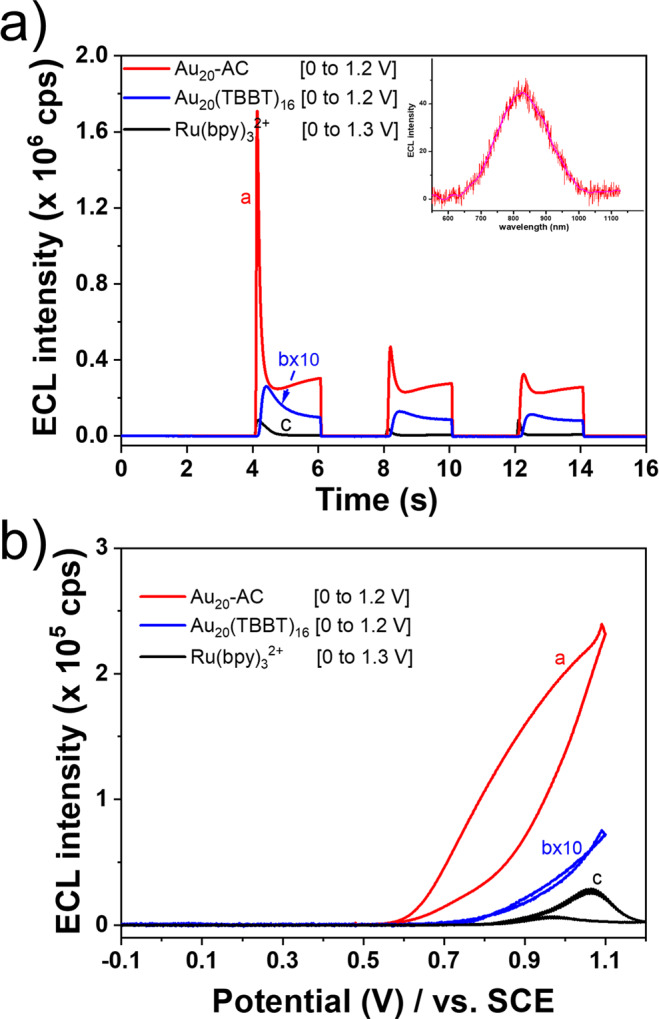
Coreactant ECL of Au_20_-AC, Au_20_(TBBT)_16_ and Ru(bpy)_3_^2+^ in solution state. Coreactant ECL of Au_20_-AC, Au_20_(TBBT)_16_ and Ru(bpy)_3_^2+^ in solution state. Coreactant ECL experiments were executed in 1:1 TOL: ACN with 0.1 M TBAP and 5 mM TPrA. Pt mesh was used as working electrode, Pt foil and SCE were used as counter and reference electrodes, respectively. **a** Potential step coreactant ECLs of Au_20_-AC, Au_20_(TBBT)_16_ and Ru(bpy)_3_^2+^ in solution state. Inset is the spectrum of Au_20_-AC in solution state. The electrode potential was held for 2 s at denoted potentials in each step over three cycles. No potential was applied in the first and final 2 s. **b** ECL-voltage curves of Au_20_-AC, Au_20_(TBBT)_16_ and Ru(bpy)_3_^2+^ in solution state. Potential scan rate is 0.1 V/s. The 3^rd^ cycle data was shown.

The ECL efficiency of Au_20_-AC and Au_20_(TBBT)_16_ is assessed by comparing to Ru(bpy)_3_^2+^-TPrA under the same measurement conditions. Figure 4a shows the step ECLs of Au_20_-AC, Au_20_(TBBT)_16_ and Ru(bpy)_3_^2+^ in solution state in the prescnece of 5 mM TPrA as coreactant, in which “catalytic route” reactions occured^42^. The ECL efficiencies of Au_20_-AC and Au_20_(TBBT)_16_ were evaluated by Ru(bpy)_3_^2+^/TPrA standard (Supplementary Fig. 16 and Table 5). The ECL efficiency of Au_20_-AC/TPrA is more than 7 times higher than that of Ru(bpy)_3_^2+^/TPrA. Intense coreactant ECL of Au_20_-AC allow its ECL spectrum to be collected. As shown the inset in Fig. 4a, Au_20_-AC display an ECL emission band centered at 830 nm. In ECL-voltage curves, the ECL signal of Au_20_-AC began to appear at about 0.5 V, and reached maximum at 1.1 V, while the signal of Au_20_(TBBT)_16_ is still very weak (Fig. 4b).

### Solid-state ECL of Au_20_-AC and Au_20_(TBBT)_16_ in coreaction pathway

**Fig. 5:**
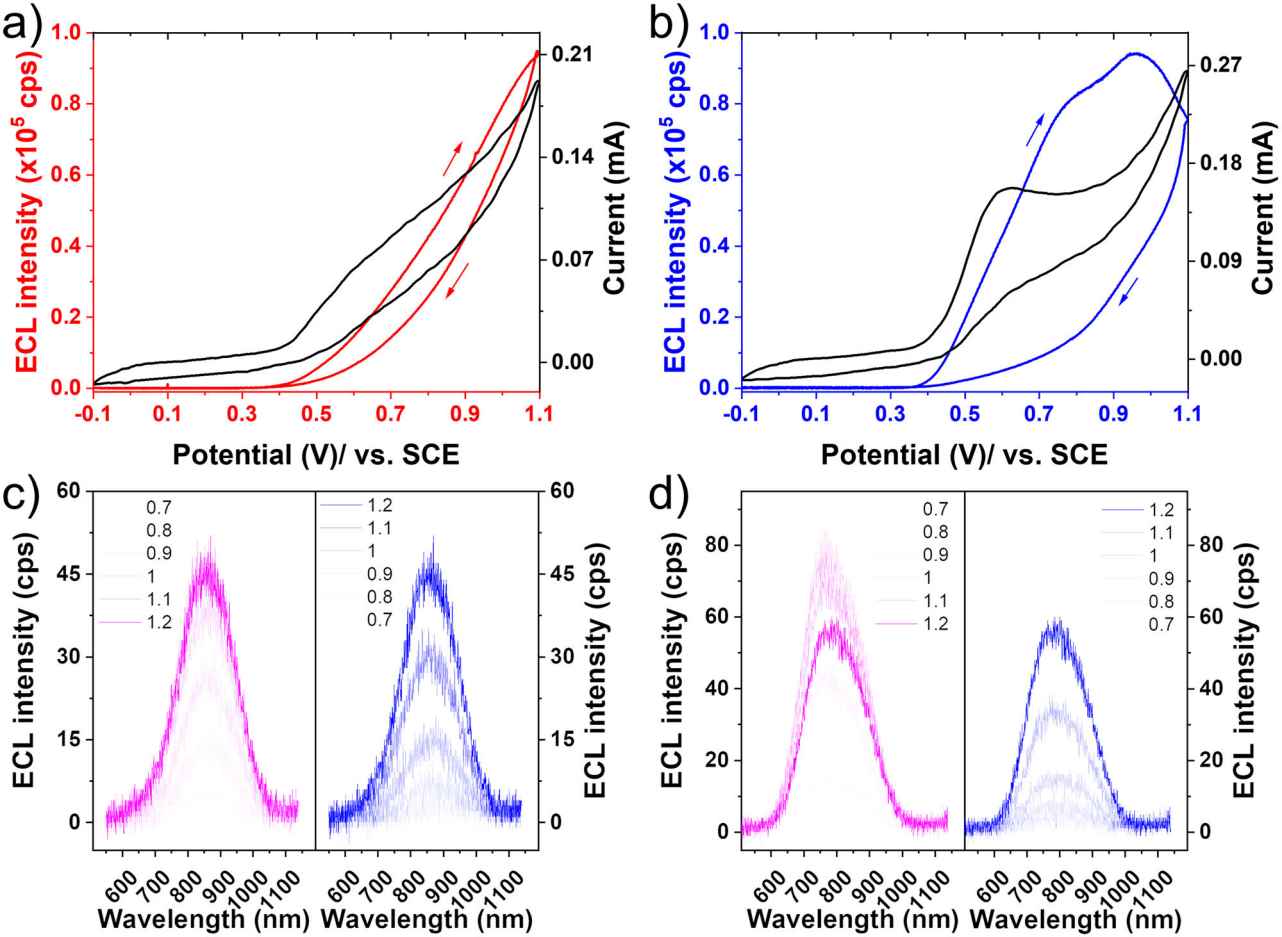
Coreactant ECLs of Au_20_-AC, Au_20_(TBBT)_16_ in solid state and their associated ECL spectra. **a**, **b** SSECL-voltage curves of Au_20_-AC (red line) and Au_20_(TBBT)_16_ (blue line) in the presence of 5 mM TPrA. Coreactant SSECL experiments were carried out in 0.01 M PBS with 0.1 M KCl. 15 μg Au_20_-AC and Au_20_(TBBT)_16_ were coated on GCE, Pt foil and SCE were used as counter and reference electrode. The potential was set from -0.1 to 1.1 V at 0.1 V/s. The 3^rd^ cycle data was shown. **c**, **d** The SSECL spectra of Au_20_-AC and Au_20_(TBBT)_16_ in the forward scan (pink) and reverse scan (blue). The potential was set from −0.1 to 1.1 V at 0.1 V/s with one spectrum collected every 1 s.

Au_20_-AC and Au_20_(TBBT)_16_ displayed red emission in solid state after light irradiation due to the AIE effect. With this in hand, we studied their SSECL. The SSECL of both nanoclusters was studied by separately loading 15 μg of each onto a GCE and allowing the electrode surface to dry in air. The electrochemical impedance spectroscopy (EIS) of both nanoclusters showed that their resistances are comparable (Supplementary Fig. 17), indicating a similar amount of both nanoclusters on electrode. The SSECL was conducted in 0.01 M phosphate buffer solution (PBS, pH = 7.5) in the presence of 0.1 M KCl as an electrolyte. As shown in Fig. 5, the similar oxidation potential of the cluster and coreactant is favorable to the ECL generation. Au_20_-AC showed the strongest emission at 1.1 V (Fig. 5a), but Au_20_(TBBT)_16_ showed the strongest emission at 0.9 V in forward scan (Fig. 5b). The relatively strong SSECL of Au_20_(TBBT)_16_ may be due to the motif aggregation effect. The long eight-membered ring motif of Au_20_(TBBT)_16_ induces intense SSECL. The SSECLs of both Au_20_ nanoclusters are much higher than that of Au_25_(SC_2_H_4_Ph)_18_ whose sweeping potential ECL cannot be detected, and step ECL is quite weak (Supplementary Fig. 18). Both RIR and coreactant effects contribute significantly to the intense ECL performance. Intense SSECLs allow the spectra of Au_20_-AC and Au_20_(TBBT)_16_ to be collected. The ECL spectra of Au_20_-AC and Au_20_(TBBT)_16_ at various potentials are presented in Fig. 5c, d. No emission peak shift was observed for either Au_20_ cluster. All the peaks of Au_20_-AC were centered at λ_max_ = 860 nm, and the strongest emission of Au_20_(TBBT)_16_ was at λ_max_ = 770 nm. Therefore, the ECL emission of Au_20_-AC was 15 nm red-shifted compared to its PL emission, but the ECL emission of Au_20_(TBBT)_16_ was 20 nm blue-shifted compared to its PL emission. This slight difference between PL and ECL might reflect differences in their excited states^43^.

## Conclusions

The structurally predicted Au_20_(SAdm)_12_(CHT)_4_ (Au_20_-AC) nanocluster has been prepared and its electrochemiluminescence performance compared with that of its isomer Au_20_(TBBT)_16_. Au_20_-AC showed a strongly cathodic and weakly anodic ECL via a self-annihilation pathway in solution state. By comparing the averaged Au-Au and Au-S distances in various gold cluster radicals, DFT simulation revealed the impact of the gold core on the electrochemiluminescence of Au_20_-AC and its isomer Au_20_(TBBT)_16._ The [Au_20_-AC]^-∙^ with a deformed Au_7_ kernel are metastable, which results in its weakly anodic ECL. Solid Au_20_-AC and Au_20_(TBBT)_16_ both showed intense ECL in the presence of the TPrA co-reactant, and therefore are suitable for developing solid-state, anti-quenching ECL luminophores. Coreactant SSECL spectra were also collected, and their intense signals enabled ECL and PL to be compared. Further work to explore and address the interactions between the emitter, electrode, and solution interfaces are underway. This work provides insights into the relationship between the structure and ECL properties of atomic precision metal nanoclusters, and is expected to pave the way for new emitters that can be used for biosensing and immunoassays.

## Materials and methods

### Synthesis

Au_20_-AC is prepared by ligand etching using Au_18_(CHT)_14_ as a precursor. Au_18_(CHT)_14_ nanoclusters were synthesized according to the method reported in the literature^39^.

Step 1: Synthesis of Au_18_(CHT)_14_. 150 mg HAuCl_4_·3H_2_O and 260 mg L‐glutathione were mixed with methanol for 15 min and then stirred at a lower speed. After 15 min, the mixture was diluted with a large amount (20 mL) of methanol, with high-speed stirring. After 15 min, a freshly prepared solution of sodium cyanoborohydride (NaBH_3_CN, 63 mg in 5 ml methanol) was added. After 6 h, the methanol solution was dried and 20 mL H_2_O and 20 mL dichloromethane (CH_2_Cl_2_) added. Excess cyclohexanethiol (1 mL) was added at 40 °C, to accomplish the two-phase ligand exchange. After stirring for 10 hours, the dichloromethane phase was dried and the Au_18_(SC_6_H_11_)_14_ residue thoroughly washed with methanol.

Step 2: Synthesis of Au_20_(SAdm)_12_(CHT)_4_. 100 mg (598.80 μmol) 1-Adamantanethiol (HSAdm) was added to a solution of Au_18_(SC_6_H_11_)_14_ (~10 mg, 1.94 μmol) in CH_2_Cl_2_ at 40 °C. After mixing overnight, the mixture was pipetted onto a TLC plate which was separated in a developing tank (CH_2_Cl_2_/Hex =1:4 v/v) (Supplementary Fig. 1). Bands corresponding to the different nanoclusters were removed and dissolved in CH_2_Cl_2_. Rhombic crystals of Au_20_(SAdm)_12_(CHT)_4_ were obtained within 3 days via layer diffusion of methanol into a CH_2_Cl_2_ solution of the nanoclusters. The yield was about 10.2% based on the Au element (calculated from HAuCl_4_·3H_2_O).

### Electrochemical measurements

Electrochemical experiments were performed on a CHI 660e. A platinum mesh was used as the working electrode. A Pt foil and saturated calomel electrode (SCE) served as the counter and reference electrodes, respectively. The concentration of the samples was ~0.015 mM with 0.1 M TBAP in 10 ml toluene/acetonitrile (Volume ratio 1:1), and the solution was purged with argon for 15 min before experiments. All data were collected at room temperature.

ECL experiments were also performed with a three-electrode system in a quartz cuvette. A platinum mesh as the working electrode, which can provide a larger specific surface area compared to platinum disk electrodes. The dimensions of the platinum mesh electrodes used in the experiments are all 10 × 10 mm, and the effective surface area is 92.9 mm^2^ according to the calculation formula $I=2.69\times {10}^{5}A{D}^{1/2}{n}^{3/2}{v}^{1/2}c$^44^. The cuvettes were aligned at a fixed position with respect to the camera for consistency. 15 μg samples were coated on glassy carbon electrode (GCE) for SSECL. A saturated calomel electrode (SCE) served as the reference electrode and a Pt foil as the counter electrode. The emission intensity was recorded with an Andor iDUS CCD camera (model No: DU401A-BR-DD). The camera was externally triggered by the potentiostat (Gamry Reference 600+) for synchronization. ECL spectra were collected with an Andor spectrograph (Kymera 193i). The sample solution was purged for about 15 min with argon prior to the measurements.

## Supplementary information

Supplementary Information

Description of Additional Supplementary Files

Supplementary Data 1

## Data Availability

The data that support the findings of this study, including supplementary method, figures, and tables, are available in its Supplementary Information files. Other relevant data are available from the corresponding author upon reasonable request. The X-ray crystallographic coordinates for structures of Au_20_(SAdm)_12_(CHT)_4_ in this article can be found from Supplementary Data 1 or at the Cambridge Crystallographic Data Centre (CCDC: www.ccdc.cam.ac.uk) under accession number 2120320.
