# Supplementary material for: Impact of the metal core on the electrochemiluminescence of a pair of atomically precise Au20 nanocluster isomers
Source: Commun Chem. 2023 May 31;6:105. doi: 10.1038/s42004-023-00907-4 (PMC10232509; doi:10.1038/s42004-023-00907-4)
Supplement: Supplementary file 1 — Supplementary Information [file 42004_2023_907_MOESM1_ESM.pdf]

**Supporting Information**

# Impact of the metal core on the electrochemiluminescence of a pair of atomically precise $Au_{20}$ nanocluster isomers

Shuang Chen,[1,2,3,4, §,*] Ying Liu,[1,2.3.4,§] Kaiyang Kuang, [1,2.3.4,§] Bing Yin, [1,2.3.4] Xiaojian Wang, [1,2.3.4] Lirong Jiang, [1,2.3.4] Pu Wang,[5,6*] Yong Pei,[5,6*] Manzhou Zhu[1,2.3.4,*]

1. Institutes of Physical Science and Information Technology, Anhui University, Hefei, Anhui 230601, P. R. China.
2. Centre for Atomic Engineering of Advanced Materials, Anhui University, Hefei, Anhui 230601, P. R. China.
3. Key Laboratory of Structure and Functional Regulation of Hybrid Materials of Ministry of Education, Anhui University, Hefei, Anhui 230601, P. R. China.
4. Department of Chemistry and Anhui Province Key Laboratory of Chemistry for Inorganic/Organic Hybrid Functionalized Materials, Anhui University, Hefei, Anhui 230601, P. R. China.
5. Department of Chemistry, Xiangtan University, Xiangtan, Hunan 411105, P. R. China
6. Key Laboratory of Environmentally Friendly Chemistry and Applications of Ministry of Education, Xiangtan University, Xiangtan, Hunan 411105, P. R. China

* Correspondence author: chenshuang@ahu.edu.cn; 90wangpu@xtu.edu.cn; ypnku78@gmail.comd; zmz@ahu.edu.cn.

## Section 1. Supplementary Methods

### Materials

Unless specified, all reagents were purchased from Sigma-Aldrich and used as received without further purification. Tetrachloroauric (III) acid ($HAuCl_4 \cdot 3H_2O$, 99.99% metal basis), sodium cyanoborohydride ($CH_3BNNa$, 99.99% metal basis, Aldrich), 1-adamantanethiol (HS-Adm, >99%), Cyclohexanethiol(CHT, >99%), dichloromethane (DCM, HPLC grade, ≥99.9%), n-hexane (Hex, HPLC grade, ≥99.9%), methanol (MeOH, HPLC grade, ≥99.9%), toluene (TOL, HPLC grade, ≥99.9%), acetonitrile (ACN, HPLC grade, ≥99.9%), tetrabutylammonium perchlorate (TBAP, >98%), tripropylamine (TPrA, >99%), thin layer chromatography (TLC) plates (iChromatography, silica gel, 250Um), were used as received. All glassware was cleaned with aqua regia (HCl: $HNO_3$=3:1 V:V), washed with copious amounts of distilled water, and dried in an oven prior to use.

### Instrumentations

**Electrospray ionization mass spectrometry.** A dilute solution of the $Au_{20}$ crystals in a mixture of DCM and MeOH was prepared and centrifuged for 5 minutes (9000 rpm) to remove any insoluble material. The centrifuged solution was then injected into a Waters Xevo G2-XS Q Tof mass spectrometer at a flow rate 5 μL/min. The source temperature was maintained at 70 °C. The results were analyzed in positive ionization modes of the ESI-MS.

**X-ray photoelectron spectroscopy.** X-ray photoelectron spectroscopy (XPS) measurements were performed on a Thermo ESCALAB 250, configured with a monochromated Al Kα (1486.8 eV) 150 W X-ray source, 0.5 mm circular spot size, a flood gun to counter charging effects, and equipped with an analysis chamber whose base pressure was lower than $1x10^{-9}$ mbar. Data were collected at FAT = 20 eV.

**Thermogravimetric analysis.** Thermogravimetric analysis (TGA) was carried out on a thermogravimetric analyzer (TGA Q5000 V3.17 Build 265) with ~6 mg of $Au_{20}$ in an Alumina ($Al_2O_3$) pan at a heating rate of 10 °C/min from room temperature to 800 °C, respectively.

**UV-visible absorption spectroscopy.** The UV-Vis absorption spectrum of $Au_{20}$ dissolved in DCM were recorded using Agilent 8453 diode array spectrometer. The background correction was made using a DCM blank. Solid samples were dissolved in DCM to make a dilute solution, with a subsequent transformation to a 1 cm path length quartz cuvette, followed by spectral measurements.

**Photoluminescence spectroscopy.** Photoluminescence spectra were measured on an Andor spectrograph (Kymera 193i). The same amount of solid sample of $Au_{20}$-AC and $Au_{20}(TBBT)_{16}$ were fixed in a quartz cell, and 410 nm light were used to excite the samples. For the solution of $Au_{20}$-AC, the sample was dissolved in DCM and diluted (OD = ~0.05).

**Single-crystal X-ray diffraction analyses.** The data collection for single crystal X-ray diffraction was carried out on a Bruker D8 venture diffractometer at 160 K, using a Cu-$K_\alpha$ radiation ($\lambda$ = 1.54186 Å) for $Au_{20}$-AC. Data reduction and absorption corrections were performed using the SAINT and SADABS programs,[1] respectively. The structure was solved by direct methods (SHELXS) and refined with full-matrix least squares on $F^2$ using the OLEX. The solvent was squeezed by platon, due to large solvent voids.[2,3] The refinement parameters are summarized in Table S1.

**Computational method and details:** DFT calculations were performed to optimize the neutral, anion and cation $Au_{20}$ clusters. The generalized gradient approximation (GGA) and Perdew-Burke-Ernzerhof (PBE) functional were used in the calculation.[4] Double-ζ numerical basis set with polarization d-function (DND) and Semi-core Pseudopot (DSPP) approximation were used to treat the atomic orbitals and core electrons, respectively.[5] In the calculation, all the $Au_{20}$ clusters used real ligands. The Tkatchenko-Scheffler (TS)[6] method was used in the dispersion correction. All the calculations were performed using the DMol3 software.[7-8]

Time-dependent density functional theory (TDDFT) calculations were performed with the Amsterdam Density Functional (ADF 2016) package.[9] The BP86[10-11] exchange-correlation functional and an all-electron STO-type basis set of double-ζ plus polarization (DZP) were used for excited state optimization and emission spectrum calculations. Scalar relativistic effects were included by utilizing the zeroth-order regular approximation (ZORA).[12] BP86 has been previously employed successfully in excited state optimization calculations of gold and silver nanoclusters.[13-14] The energy and gradient convergence criteria were tightened to $1 \times 10^{-4}$ and $1 \times 10^{-3}$ to obtain well-converged geometries.

## Section 2. Supplementary Figures

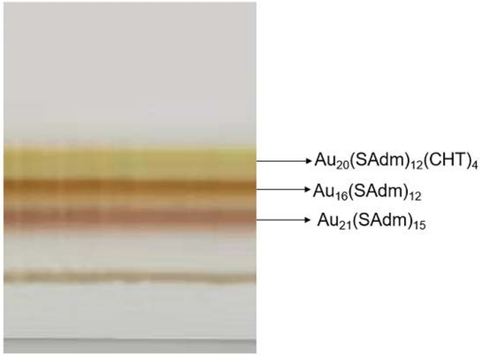


**Supplementary Fig. 1** Photograph of the raw product separated by TLC plate.

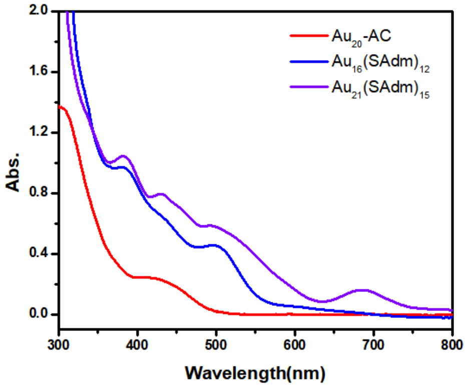


**Supplementary Fig. 2** UV-Vis spectra of $Au_{20}(SAdm)_{12}(CHT)_4$, $Au_{16}(SAdm)_{12}$, and $Au_{21}(SAdm)_{15}$ separated from TLC plate.

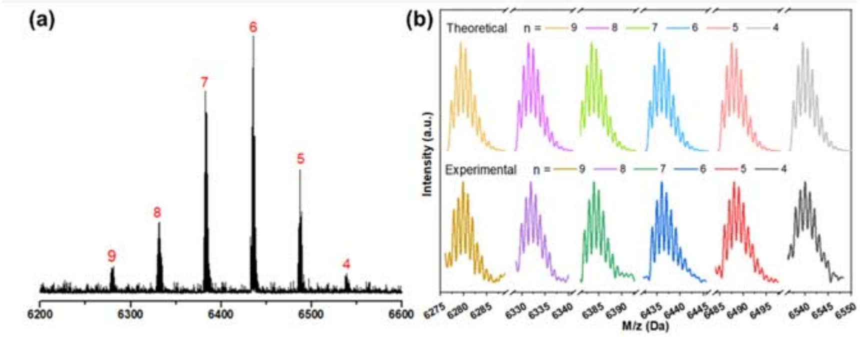


**Supplementary Fig. 3** (a) ESI-MS (positive ion mode) spectrum of $Au_{20}$ nanocluster in DCM/MeOH with cesium acetate (CsOAc) added. The red labeled are $[Au_{20}(C_{10}H_{15}S)_{16-n}(C_6H_{11}S)_n Cs]^+$ (n=4~9). (b) Theoretical and experimental isotopic peaks.

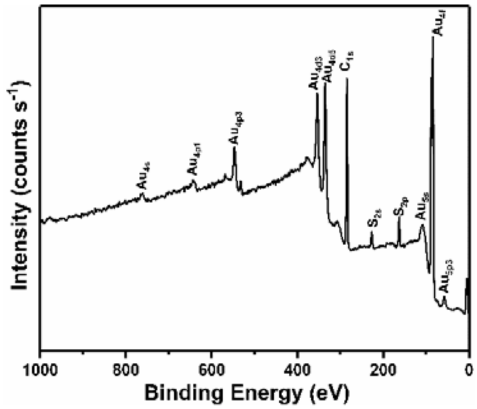


**Supplementary Fig. 4** The X-ray photoelectron spectroscopy (XPS) of $Au_{20}(SAdm)_{12}(CHT)_4$.

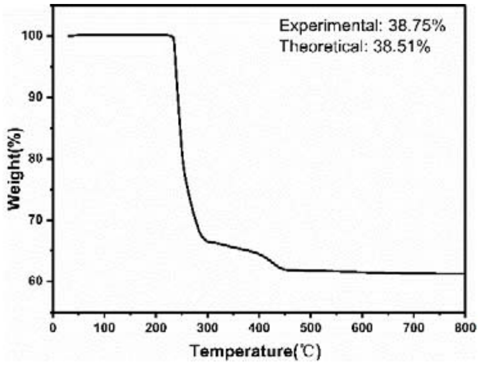


**Supplementary Fig. 5** The thermogravimetric analysis (TGA) of $Au_{20}(SAdm)_{12}(CHT)_4$.

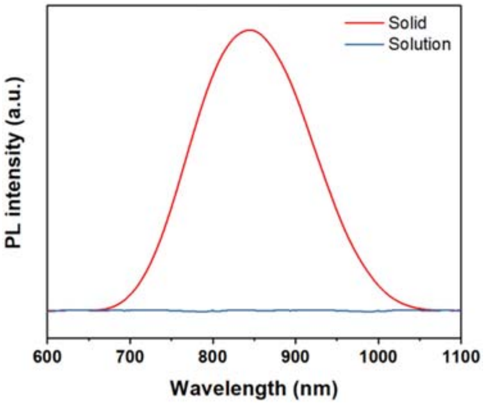


**Supplementary Fig. 6** The aggregated $Au_{20}$-AC has obvious photoluminescence, while $Au_{20}$-AC display extremely weak and negligible emission in DCM solution.

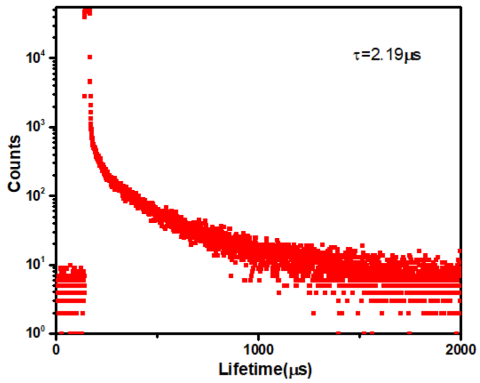


**Supplementary Fig. 7** PL decay profiles (excitation wavelength 367nm; recorded at the corresponding emission maximum) of the $Au_{20}$-AC.

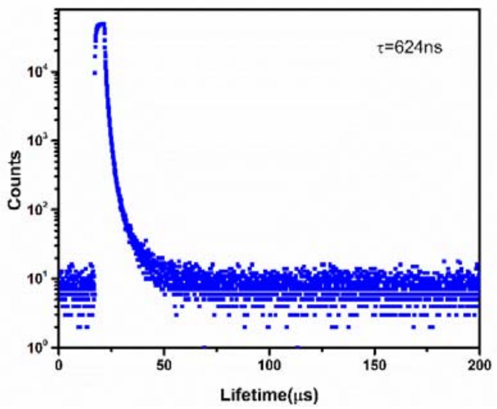


**Supplementary Fig. 8** PL decay profiles (excitation wavelength 367nm; recorded at the corresponding emission maximum) of the $Au_{20}(TBBT)_{16}$.

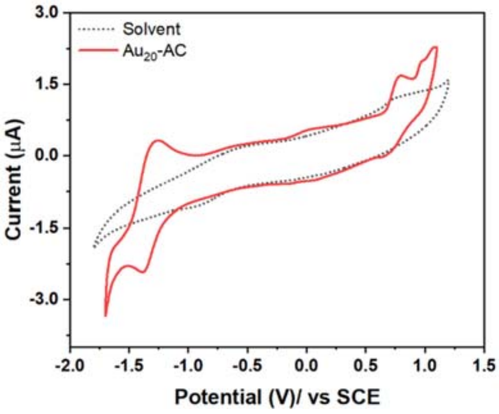


**Supplementary Fig. 9** Cyclic voltammogram curves of $Au_{20}$-AC in 1:1 TOL: ACN

with 0.1 M TBAP from -1.7 V to 1.1 V. Potential scan rate is 0.1 V/s.

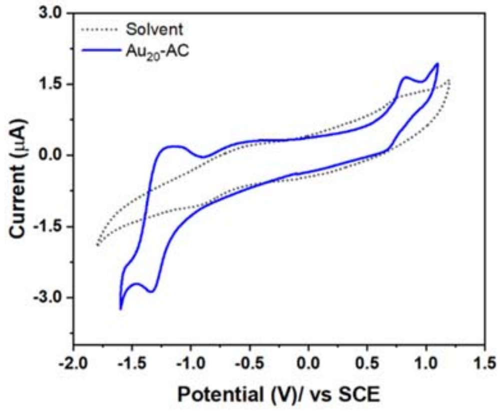


**Supplementary Fig. 10** Cyclic voltammogram curves of $Au_{20}(TBBT)_{16}$ in 1:1 TOL: ACN with 0.1 M TBAP from -1.6 V to 1.1 V. Potential scan rate is 0.1 V/s.

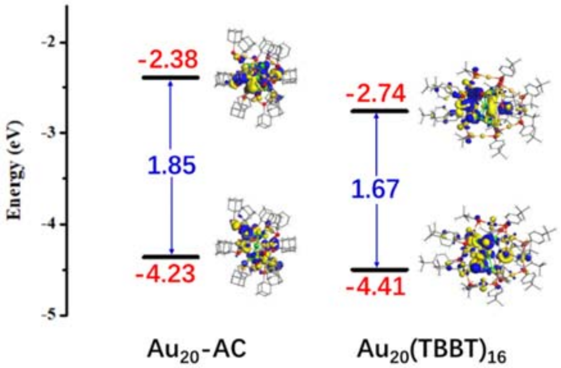


**Supplementary Fig. 11** The contour plots and energy levels of $Au_{20}$-AC and $Au_{20}(TBBT)_{16}$ clusters. All the isovalues for the MOs plots are 0.02.

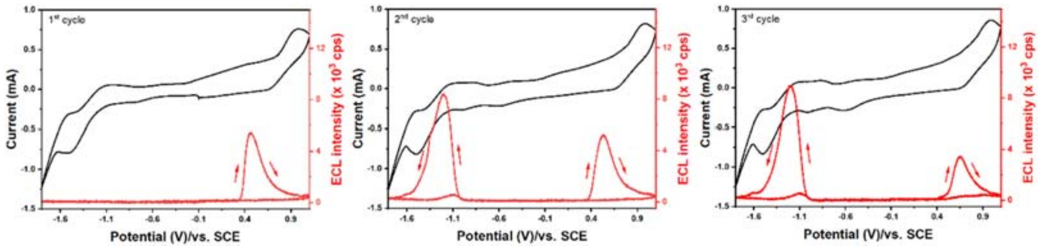


Supplementary **Fig. 12** The CV and ECL of $Au_{20}$-AC over three cycles under consecutive

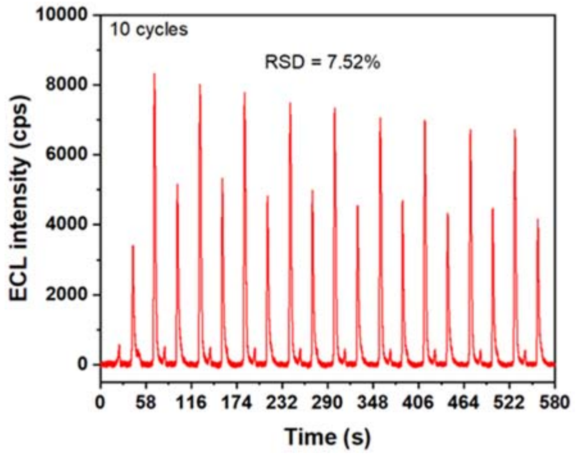


Supplementary **Fig. 13** The stability of Au20-AC under consecutive potential scans.

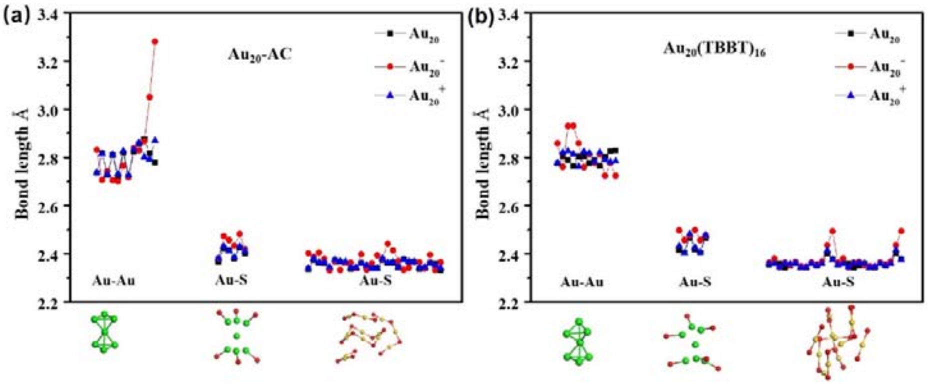


**Supplementary Fig. 14** Summarization of bond length Au-Au (in metal core) and Au-S (in core-shell and motif) for both Au20 nanoclusters, the corresponding averaged bond length is in Table S3.

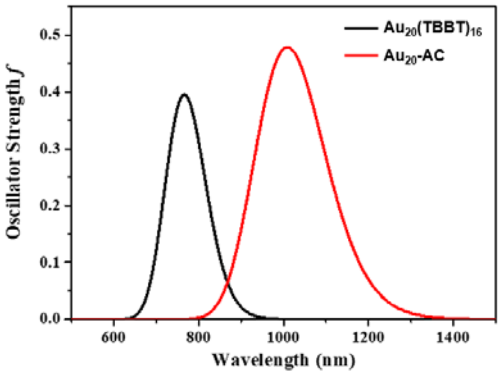


**Supplementary Fig. 15** The oscillator strength of Au20 isomers in the emission spectrum consistent with Table S4.

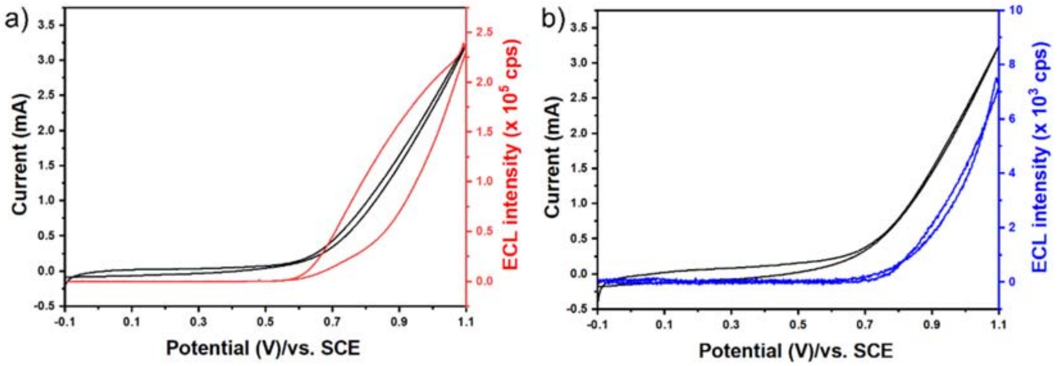


**Supplementary Fig. 16** CV (left axis) and ECL-potential (right axis) curves of both $Au_{20}$ nanoclusters with 5 mM TPrA. For CV and ECL measurements, a Pt mesh was used as working electrode in a 20 mL cuvette and purging with Ar. The purging process is continued to 20 min before test. The concentration of nanocluster is ~0.015 mM. The supporting electrolyte is 0.1 M TBAP. Potential scan rate is 0.1 V/s.

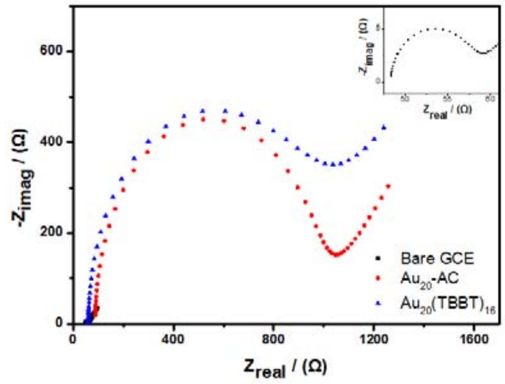


**Supplementary Fig. 17** Nyquist plots of bare GCE, $Au_{20}$-AC/$Au_{20}(TBBT)_{16}$ on GCE in 0.01 M PBS containning 0.1 M KCl and 0.01 M $[Fe(CN)_6]^{3-/4-}$.

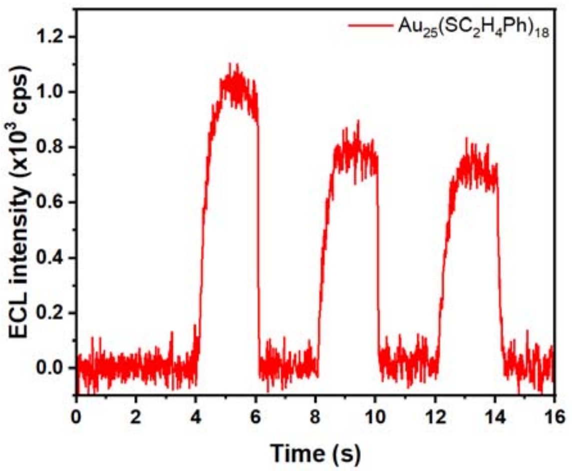


**Supplementary Fig. 18** The step SSECL of $Au_{25}(SC_2H_4Ph)_{18}$ in the presence of 5 mM TPrA as coreactant. The SSECL experiment was carried out in 0.01 M PBS with 0.1 M KCl. 15 μg $Au_{25}(SC_2H_4Ph)_{18}$ were coated on GCE, Pt foil and SEC were used as counter and reference electrode. The electrode potential was held for 2 s from 0 to 1.1 V in each step over three cycles. No potential was applied in the first and final 2 s.

**Section 3. ECL mechanism**

A possible self-annihilation ECL mechanism of the $Au_{20}$ clusters is proposed in Supplementary Equation (1-4):

(1) $Au_{20} + e^{-} \rightarrow Au_{20}^{-\bullet}$

(2) $Au_{20} \rightarrow Au_{20}^{+\bullet} + e^{-}$

(3) $Au_{20}^{+\bullet} + Au_{20}^{-\bullet} \rightarrow Au_{20}{*} + Au_{20}$

(4) $Au_{20}{*} \rightarrow Au_{20} + h\nu$

A possible mechanism for the ECL of $Au_{20}$-AC and $Au_{20}(TBBT)_{16}$ (unified abbreviated as $Au_{20}$) with TPrA is shown below. An oxidation reaction occurs on the electrode surface to produce $TPrA^{+\bullet}$ and $Au_{20}^{+\bullet}$ (Supplementary Equation (5 and 6)). The $TPrA^{+\bullet}$ radical cation is deprotonated (loss of $H^{+}$) to generate the strongly reducing free radical $TPrA^{\bullet}$ (Supplementary Equation (7)), which migrates to the electrode/cluster solid interface and react with $Au_{20}^{+\bullet}$ to form the excited species $Au_{20}{*}$ (Supplementary Equation (8)), which relaxes to the ground state and emits light (Supplementary Equation (9)).

(5) $TPrA \rightarrow TPrA^{+\bullet} + e^{-}$

(6) $Au_{20} \rightarrow Au_{20}^{+\bullet} + e^{-}$

(7) $TPrA^{+\bullet} \rightarrow TPrA^{\bullet} + H^{+}$

(8) $Au_{20}^{+\bullet} + TPrA^{\bullet} \rightarrow Au_{20}{*} + Pr_2N^{+}{=}CHCH_2CH_3$

(9) $Au_{20}{*} \rightarrow Au_{20} + h\nu$

## Section 4. Supplementary Tables

**Supplementary Table 1.** Crystal data and structure refinement for the $Au_{20}(SAdm)_{12}(CHT)_4$ nanocluster.

| | |
|---|---|
| Identification code | $Au_{20}(SAdm)_{12}(CHT)_4$ |
| Empirical formula | $C_{144}$ $H_{224}$ $Au_{20}$ $S_{16}$ |
| Formula weight | 6406.64 |
| Temperature/K | 160 |
| Crystal system | monoclinic |
| Space group | C2/c |
| a/Å | 27.6893(15) |
| b/Å | 17.8685(11) |
| c/Å | 37.389(2) |
| α/° | 90 |
| β/° | 109.308(4) |
| γ/° | 90 |
| Volume/Å3 | 17458.2(18) |
| Z | 1 |
| ρcalcg/cm3 | 0.023 |
| μ/mm 1 | 0.412 |
| F(000) | 102.0 |
| Radiation | CuKα (λ = 1.54186) |
| 2Θ range for data collection/° | 7.058 to 139.482 |
| Index ranges | $-33 \leq h \leq 22$, $-21 \leq k \leq 17$, $-39 \leq l \leq 45$ |
| Reflections collected | 77956 |
| Independent reflections | 16123 [$R_{int}$ = 0.0590, $R_{sigma}$ = 0.0372] |
| Data/restraints/parameters | 16123/1852/822 |
| Goodness-of-fit on F2 | 1.053 |
| Final R indexes [I>=2σ (I)] | $R_1$ = 0.0900, $wR_2$ = 0.2467 |
| Final R indexes [all data] | $R_1$ = 0.1029, $wR_2$ = 0.2583 |
| Largest diff. peak/hole / e Å-3 | 4.62/-2.34 |

**Supplementary Table 2.** The Au-Au bond length of $Au_7$ core in $Au_{20}$-AC and $Au_{20}(TBBT)_{16}$ with different valence states.

| | **$Au_{20}$-AC** | | | **$Au_{20}(TBBT)_{16}$** | | |
|---|---|---|---|---|---|---|
| **Au-Au bond length** | **$Au_{20}$** | **$Au_{20}^{-}$** | **$Au_{20}^{+}$** | **$Au_{20}$** | **$Au_{20}^{-}$** | **$Au_{20}^{+}$** |
| **Au1-Au2** | 2.857 | 2.838 | 2.862 | 2.829 | 2.761 | 2.820 |
| **Au1-Au3** | 2.818 | 2.831 | 2.816 | 2.804 | 2.930 | 2.827 |
| **Au2-Au3** | 2.810 | **3.281** | 2.812 | 2.764 | 2.859 | 2.777 |
| **Au5-Au6** | 2.819 | **3.050** | 2.827 | 2.827 | 2.759 | 2.824 |
| **Au6-Au7** | 2.823 | 2.829 | 2.832 | 2.805 | 2.931 | 2.813 |
| **Au5-Au7** | 2.876 | 2.867 | 2.870 | 2.766 | 2.859 | 2.762 |
| **Au4-Au1** | 2.737 | 2.707 | 2.737 | 2.776 | 2.776 | 2.782 |
| **Au4-Au2** | 2.728 | 2.706 | 2.730 | 2.804 | 2.813 | 2.818 |
| **Au4-Au3** | 2.818 | 2.743 | 2.793 | 2.790 | 2.724 | 2.785 |
| **Au4-Au5** | 2.714 | 2.700 | 2.732 | 2.803 | 2.814 | 2.820 |
| **Au4-Au6** | 2.779 | 2.766 | 2.801 | 2.776 | 2.777 | 2.782 |
| **Au4-Au7** | 2.720 | 2.718 | 2.725 | 2.789 | 2.724 | 2.790 |

**Supplementary Table 3.** Summarization of averaged Au-Au and Au-S distances in various gold clusters.

| Layers(Å)[a] / Au Clusters | Au-Au ($Au_7$ core) | Au-S (core-shell) | Au-S (motif) |
|---|---|---|---|
| $Au_{20}$-AC | 2.792 | 2.402 | 2.355 |
| $[Au_{20}\text{-AC}]^{-}$ | 2.837 | 2.441 | 2.370 |
| $[Au_{20}\text{-AC}]^{+}$ | 2.795 | 2.409 | 2.358 |
| $Au_{20}(TBBT)_{16}$ | 2.794 | 2.430 | 2.357 |
| $Au_{20}(TBBT)_{16}^{-}$ | 2.810 | 2.478 | 2.376 |
| $Au_{20}(TBBT)_{16}^{+}$ | 2.800 | 2.438 | 2.361 |

**Supplementary Table 4.** The emission energy and oscillator strength in the calculated PL emission spectrum.

| **Level** | **Au clusters** | **$E_{em}$ (eV)** | **f (oscillator strength)** |
|---|---|---|---|
| **BP86/DZP $S_1 \rightarrow S_0$** | $Au_{20}$-AC | 1.23 | $1.2\times10^{-2}$ |
| | $Au_{20}(TBBT)_{16}$ | 1.62 | $9.9\times10^{-3}$ |

**Supplementary Table 5.** ECL efficiencies of $Au_{20}$-AC and $Au_{20}(TBBT)_{16}$ nanoclusters.

| | $\frac{I_{NC}}{I_{Ru(bpy)_3^{2+}}}a$ | $\frac{ECL_{NC}}{ECL_{Ru(bpy)_3^{2+}}}$ | ΦECL%[b] |
|---|---|---|---|
| $Au_{20}(TBBT)_{16}$ | 0.657 | 0.03 | 4.7 |
| $Au_{20}$-AC | 1.04 | 7.98 | 769 |

[a]IECLx means ECL intensity of x species. [b]Determined relative to that of the $Ru(bpy)_3^{2+}$/TPrA system that has an absolute ϕECL of 5%.

## Supplementary References


[1] APEX II software suite, *Bruker-AXS*, **2006**.

[2] SHELXTL, Sheldrick, G. M. *Acta Crystallogr.* **2015**, *C 71*, 3-8.

[3] Dolomanov, O.V., Bourhis, L.J., Gildea, R.J, Howard, J.A.K. & Puschmann, H., *J. Appl. Cryst.* **2009**, *42*, 339-341.

[4] Perdew, J. P.; Burke, K.; Ernzerhof, M. *Phys. Rev. Lett.* **1996**, *77*, 3865-3868.

[5] Delley, B. *J. Phys. Chem. A* **2006**, *110*, 13632-13639.

[6] Tao, J.; Perdew, J. P.; Staroverov, V. N.; Scuseria, G. E. *Phys. Rev. Lett.* **2003**, *91*, 146401.

[7] Delley, B. Delley. *J. Chem. Phys.* **1990**, *92,* 508–517.

[8] Delley, B. *J. Chem. Phys.* **2000**, *113*, 7756−7762.

[9] te Velde, G.; Bickelhaupt, F. M.; Baerends, E. J.; Fonseca Guerra, C.; van Gisbergen, S. J. A.; Snijders, J. G.; Ziegler, T.*J. Comput. Chem.* **2001**, *22*, 931−967.

[10] Becke, A. D. *Phys. Rev. A* **1988**, *38*, 3098−3100.

[11] Perdew, J. P. *Phys. Rev. B* **1986**, *33*, 8822−8824.

[12] Lenthe, E. v.; Baerends, E. J.; Snijders, J. G. *J. Chem. Phys.* **1993**, *99*, 4597−4610.

[13] Guidez, E. B.; Aikens, C. M. *J. Phys. Chem. A* **2015**, *119*, 3337−3347.

[14] Aikens, C. M. *J. Phys. Chem. A* **2009**, *113*, 10811−10817.
