# Supplementary material for: Impact of the metal core on the electrochemiluminescence of a pair of atomically precise Au20 nanocluster isomers
Source: Commun Chem. 2023 May 31;6:105. doi: 10.1038/s42004-023-00907-4 (PMC10232509; doi:10.1038/s42004-023-00907-4)
Supplement: Supplementary file 2 — Description of Additional Supplementary Files [file 42004_2023_907_MOESM2_ESM.pdf]

# Description of Additional Supplementary File

**File name:** Supplementary Data 1

**Description:** CIF for $Au_{20}(SAdm)_{12}(CHT)_4$.
